# A Comparative Evaluation of Statistical Product and Service Solutions (SPSS) and ChatGPT-4 in Statistical Analyses

**DOI:** 10.7759/cureus.72581

**Published:** 2024-10-28

**Authors:** Al Imran Shahrul, Alizae Marny F Syed Mohamed

**Affiliations:** 1 Department of Family Oral Health, Faculty of Dentistry, Universiti Kebangsaan Malaysia, Kuala Lumpur, MYS

**Keywords:** artificial intelligence, data analysis, machine learning, natural language processing, software comparison, statistical software

## Abstract

Background: The objective of this study was to assess the accuracy of Chat Generative Pre-trained Transformer 4.0 (ChatGPT-4; OpenAI, San Francisco, CA ) compared to Statistical Product and Service Solutions (SPSS; IBM SPSS Statistics for Windows, Armonk, NY) in performing statistical analyses commonly used in medical and dental research.

Methods: The datasets were analysed using SPSS (version 26) and ChatGPT-4. Statistical tests included the independent t-test, paired t-test, ANOVA, chi-square test, Wilcoxon signed-rank test, Mann-Whitney U test, Pearson and Spearman correlation, regression analysis, kappa statistic, intraclass correlation coefficient (ICC), Bland-Altman analysis, and sensitivity and specificity analysis. Descriptive statistics were used to report results, and differences between the two tools were noted.

Results: SPSS and ChatGPT-4 produced identical results for the independent sample t-test, paired t-test, and simple linear regression. In one-way ANOVA, both tools provided consistent F-values, but post-hoc analysis revealed discrepancies in mean differences and confidence intervals. Pearson chi-square and Wilcoxon signed-rank tests showed variations in p-values and Z-values. Mann-Whitney U test had differences in interquartile range (IQR), U, and Z-values. Pearson and Spearman's correlations were consistent, with IQR differences in Spearman. Sensitivity, specificity, and area under the curve (AUC) analyses were consistent, though differences in standard errors and confidence intervals were observed.

Conclusion: ChatGPT-4 produced accurate results for several statistical tests, matching SPSS in simpler analyses. However, discrepancies in post-hoc analyses, confidence intervals, and more complex tests indicate that careful validation is required when using ChatGPT-4 for detailed statistical work. Researchers should exercise caution and cross-validate results with established tools such as SPSS.

## Introduction

Artificial intelligence (AI) has revolutionized data analysis by introducing new capabilities for processing large datasets and automating complex tasks [1]. This advancement is especially beneficial in fields such as medicine and dentistry, where quick and accurate data analysis is critical for decision-making. The Chat Generative Pre-trained Transformer 4.0 (ChatGPT-4), developed by OpenAI [2], is an advanced AI language model that uses machine learning techniques, particularly deep learning, to process and generate human-like text. Through training on vast datasets, ChatGPT-4 learns patterns in text, enabling it to handle a wide range of queries, from simple conversations to complex tasks such as code generation and data analysis [3].

With the release of GPT-4, ChatGPT has become even more capable, particularly in the realm of data analysis, owing to its integration with Python (Python Software Foundation, Wilmington, DE) - a programming language widely used in data science and statistics [4-6]. Python is known for its libraries, such as NumPy, pandas, and SciPy, which facilitate complex statistical operations [7]. The "Data Analyst" feature automatically responds to natural language inputs, offering a significant advancement by eliminating the need for manual input. Core functionalities of the 'Data Analyst' module include descriptive statistics, inferential statistics, regression analyses, graphical data representation, and predictive analytics. This natural language interface lowers the usability barrier for beginners, making 'Data Analyst' a potential tool for research purposes.

In contrast, Statistical Package for the Social Sciences (SPSS; IBM Corporation, Armonk, NY) is a widely used statistical software, particularly in fields such as social sciences, medicine, and dentistry [8]. SPSS offers a user-friendly interface that allows researchers to conduct a wide range of statistical analyses, from basic descriptive statistics to complex multivariate analyses. Its point-and-click functionality makes it accessible to users without extensive programming experience. SPSS also provides detailed output, ensuring transparency in statistical methods and assumptions, which is crucial for rigorous research.

ChatGPT-4's integration with Python offers researchers a flexible and automated approach to data analysis, enabling dynamic analyses and the use of advanced data science techniques [4]. However, the rise of AI in data analysis raises concerns about the consistency, transparency, and accuracy of its results compared to established tools such as SPSS. Evaluating these factors is vital to ensure AI platforms meet the rigorous standards required in research.

This study aims to compare SPSS and ChatGPT-4 in performing statistical analyses relevant to medical and dental research. By systematically evaluating the outputs of both tools across various statistical tests, this research seeks to determine whether ChatGPT-4 can provide reliable results that align with the rigorous standards of traditional statistical software.

## Materials and methods

Study design

This cross-sectional study was conducted at the Faculty of Dentistry, Universiti Kebangsaan Malaysia, Kuala Lumpur, Malaysia. The study took place from 11th July 2024 until 31st August 2024. Synthetic datasets were created for each statistical test [9]. The synthetic datasets were generated using ChatGPT-4 by providing specific prompts such as "Please create a synthetic dataset for each statistical analysis." These datasets were specifically designed to meet the assumptions and requirements of each respective test, ensuring valid and reliable analysis. The sample sizes varied depending on the type of analysis, ranging from smaller datasets of 30 samples for non-parametric tests to larger datasets of up to 100 samples for regression analysis. After generation, the datasets were reviewed by a statistician to ensure they met the assumptions and requirements of each statistical analysis.

Statistical tests included

The study aimed to assess the statistical analysis capabilities of ChatGPT and SPSS by comparing a variety of statistical tests. These included the independent t-test, which analysed two groups with continuous data, and the paired t-test, which compared two sets of paired continuous data. ANOVA was used to assess three or more groups with continuous data, while the chi-square test evaluated contingency tables with categorical data. Additionally, the Wilcoxon signed-rank test compared two sets of paired ordinal data, and the Mann-Whitney U test compared two independent groups with ordinal data. The Pearson correlation coefficient was utilised to measure the relationship between two continuous variables, while the Spearman correlation coefficient examined the relationship between two ordinal variables. Regression analysis was applied to evaluate the relationship between one dependent continuous variable and one or more independent variables, whether continuous or categorical. The kappa statistic assessed the agreement between two raters' categorical ratings, and the intraclass correlation coefficient (ICC) evaluated ratings or measurements across clusters or groups. Furthermore, Bland-Altman analysis was used to plot differences between the two methods against their averages, and the study included the calculation of sensitivity, specificity, and the area under the ROC curve (AUC) for binary classification models.

Process description

Data entry and initial tabulation were conducted using Microsoft Excel (Microsoft® Corp., Redmond, WA) for both SPSS and ChatGPT-4 analyses. Following data preparation, the synthetic datasets - generated in Excel and saved in CSV format - were imported into SPSS (version 26) for analysis. Each statistical test was performed using the appropriate SPSS procedures, ensuring adherence to standard protocols.

The same datasets were used for both SPSS and ChatGPT-4 analyses without any modifications. The CSV files were generated in Microsoft Excel and subsequently loaded into SPSS (version 26) for analysis. For ChatGPT-4, the CSV files were imported into a Python 3.9.6 environment and passed as Pandas DataFrames (Pandas version 1.5.3) for analysis. Additionally, the following Python libraries were employed: NumPy (version 1.24.0) for numerical operations, SciPy (version 1.9.3) for statistical tests (e.g., t-tests, Mann-Whitney U test), Statsmodels (version 0.13.5) for more advanced statistical models such as ANOVA and regression analysis, Matplotlib (version 3.6.3) for visualizations (e.g., box plots, Bland-Altman plots), and Seaborn (version 0.11.2) for enhanced data visualizations.

Simple instructions were provided to ChatGPT-4 for each statistical test; for example, to conduct an independent sample t-test, the command "Please conduct an Independent T-Test" was issued, and ChatGPT-4 executed the analysis accordingly. The data formatting (e.g., numeric and categorical variables) was kept consistent across both platforms to ensure identical input conditions. No transformations or manipulations were performed post-import, ensuring that the data remained consistent throughout the analysis. This allowed for a direct comparison between the outputs of SPSS and ChatGPT-4, guaranteeing the comparability of the results across both tools.

Statistical analysis

Microsoft Excel was used for data entry and tabulation. Descriptive statistics was utilized to report the results. Differences between SPSS and ChatGPT-4 were noted when the values differed after rounding the ChatGPT-4 results to match the ordinal values reported by SPSS.

## Results

For the independent sample t-test (Table 1) and paired t-test (Table 2), SPSS and ChatGPT-4 produced identical results regarding mean values, standard deviations, p-values, and 95% confidence intervals for the mean differences. No differences were observed between the tools in these analyses.

**Table 1:** Comparison of independent sample t-test between SPSS and ChatGPT-4

Group	Mean	Std. Deviation	Mean Diff.	95% CI (Lower, Upper)		t	df	P value
SPSS								
Group A	54.4286	11.0033	2.3238	-2.9044	7.5520	0.890	58	0.377
Group B	52.1048	9.1424						
ChatGPT								
Group A	54.4286	11.0033	2.3238	-2.9044	7.5520	0.890	58	0.377
Group B	52.1048	9.1424						

**Table 2:** Comparison of paired t-test between SPSS and ChatGPT-4

Group	Mean	Std. Deviation	Mean Diff.	95% CI (Lower, Upper)		t	df	P value
SPSS								
Before	49.4006	10.2637	-0.3828	-1.9591	1.1934	-0.497	29	0.623
After	49.7834	10.3697						
ChatGPT								
Before	49.4006	10.2637	-0.3828	-1.9591	1.1934	-0.497	29	0.623
After	49.7834	10.3697						

In the one-way ANOVA, both SPSS and ChatGPT-4 delivered consistent F-values, p-values, and descriptive statistics (Table 3). However, inconsistencies arose during the post-hoc analysis using Tukey HSD (Table 4). Although both tools identified the same significant pairwise differences, the mean differences reported by ChatGPT-4 did not align with those from SPSS. The order of the group comparisons and the presentation of 95% confidence intervals differed, which could lead to misinterpretation of the results.

**Table 3:** Comparison of one-way ANOVA between SPSS and ChatGPT-4 BG = Between Group; WG = Within Group

Group	Mean	Std. Deviation	95% CI (Lower, Upper)		F	df	P value
SPSS							
Group 1	46.6907	9.6643	43.0820	50.2994	11.218	2 (BG), 87 (WG)	<0.001
Group 2	56.0177	10.4586	52.1124	59.9230			
Group 3	58.5486	10.4936	54.6302	62.4669			
ChatGPT							
Group 1	46.6907	9.6643	43.0820	50.2994	11.218	2 (BG), 87 (WG)	<0.001
Group 2	56.0177	10.4586	52.1124	59.9230			
Group 3	58.5486	10.4936	54.6302	62.4669			

**Table 4:** Comparison of the post-hoc test for one-way ANOVA (Tukey HSD) between SPSS and ChatGPT-4 * Differences reported between SPSS and ChatGPT-4.

Paired Group	Mean Diff.	95% CI (Lower, Upper)		P value
SPSS				
Group 1 - Group 2	-9.3270	-15.6147	-3.0394	0.002
Group 1 - Group 3	-11.8579	-18.1455	-5.5703	<0.001
Group 2 - Group 3	-2.5309	-8.8185	3.7568	0.604
ChatGPT				
Group 1 - Group 2	9.3270*	3.0394*	15.6147*	0.002
Group 1 - Group 3	11.8579*	5.5703*	18.1455*	<0.001
Group 2 - Group 3	2.5309*	3.7568*	8.8185*	0.604

For the Pearson chi-square test (Table 5), both tools provided consistent chi-square statistics and overall conclusions. However, slight variations in the p-values were observed. Similarly, in the Wilcoxon signed-rank test (Table 6), the tools agreed on the overall conclusion and p-values, but small differences were found in the Z-values and interquartile ranges (IQRs). In the Mann-Whitney U test, consistent median values were reported for both tools (Table 7). However, differences emerged in the IQR for one of the groups, as well as in the U, p-values, and Z-values. One tool reported a positive Z-value, while the other reported a negative one. 

**Table 5:** Comparison of Pearson’s chi-square between SPSS and ChatGPT-4 * Differences reported between SPSS and ChatGPT-4.

Gender	Preferences, N (%)		X^2^	P value
	No	Yes		
SPSS				
Male	20 (40.0)	30 (60.0)	0.164 (likehood ratio)	0.685 (likehood ratio)
Female	22 (44.0)	28 (56.0)		
ChatGPT				
Male	20 (40.0)	30 (60.0)	0.041* (continuity correction)	0.839* (continuity correction)
Female	22 (44.0)	28 (56.0)		

**Table 6:** Comparison of Wilcoxon signed-rank test between SPSS and ChatGPT-4 * Differences reported between SPSS and ChatGPT-4.

Group	Median	Interquartile Range (IQR)	Z	P value
SPSS				
Before	6.50	5.00	-1.246	0.213
After	6.00	5.00		
ChatGPT				
Before	6.50	4.75*	-1.46*	0.213
After	6.00	4.75*		

**Table 7:** Comparison of Mann-Whitney U between SPSS and ChatGPT-4 * Differences reported between SPSS and ChatGPT-4.

Group	Median	Interquartile Range (IQR)	U	Z	P value
SPSS					
A	5.00	3.00	449.000	-0.015	0.988
B	4.50	4.00			
ChatGPT					
A	5.00	3.00*	451.000*	0.015*	0.994*
B	4.50	3.75*			

In both Pearson correlation (Table 8) and Spearman correlation analyses (Table 9), SPSS and ChatGPT-4 provided consistent correlation coefficients and p-values, leading to similar conclusions about the relationships between variables. However, in the Spearman correlation analysis, differences in the IQRs were noted, even though the primary statistical outputs were consistent.

**Table 8:** Comparison of Pearson’s correlation results between SPSS and ChatGPT-4

Variables	Mean	Std. Deviation	Pearson Correlation	P value
SPSS				
X	0.0121	1.0239	0.899 (Very Perfect Correlation)	<0.001
Y	-0.0439	2.1900		
ChatGPT				
X	0.0121	1.0239	0.899 (Very Perfect Correlation)	<0.001
Y	-0.0439	2.1900		

**Table 9:** Comparison of Spearman’s correlation, between SPSS and ChatGPT-4 * Differences reported between SPSS and ChatGPT-4.

Variables	Mean	Interquartile Range (IQR)	Correlation Coefficient	P value
SPSS				
X	54.5	57	0.085 (No Significant Correlation)	0.399
Y	50.5	45		
ChatGPT				
X	54.5	55*	0.085 (No Significant Correlation)	0.399
Y	50.5	43.5*		

For simple linear regression (Table 10), both tools produced identical results for regression coefficients, confidence intervals, t-values, and p-values, with no differences observed. In the kappa statistic analysis, both SPSS and ChatGPT-4 agreed on the kappa value, but a discrepancy was found in the reported p-values (Table 11).

**Table 10:** Comparison of simple linear regression between SPSS and ChatGPT-4

B	95% CI (Lower, Upper)		t	P value
SPSS				
2.849	2.664	3.033	30.694	<0.001
ChatGPT				
2.849	2.664	3.033	30.694	<0.001

**Table 11:** Comparison of kappa statistics between SPSS and ChatGPT-4

Rater	Rater 2		Kappa	P value
Rater 1	No	Yes		
SPSS				
No	24 (53.3)	21 (46.7)	0.060	0.546
Yes	26 (47.3)	29 (52.7)		
ChatGPT				
No	24 (53.3)	21 (46.7)	0.060	0.548*
Yes	26 (47.3)	29 (52.7)		

**Figure 1:**
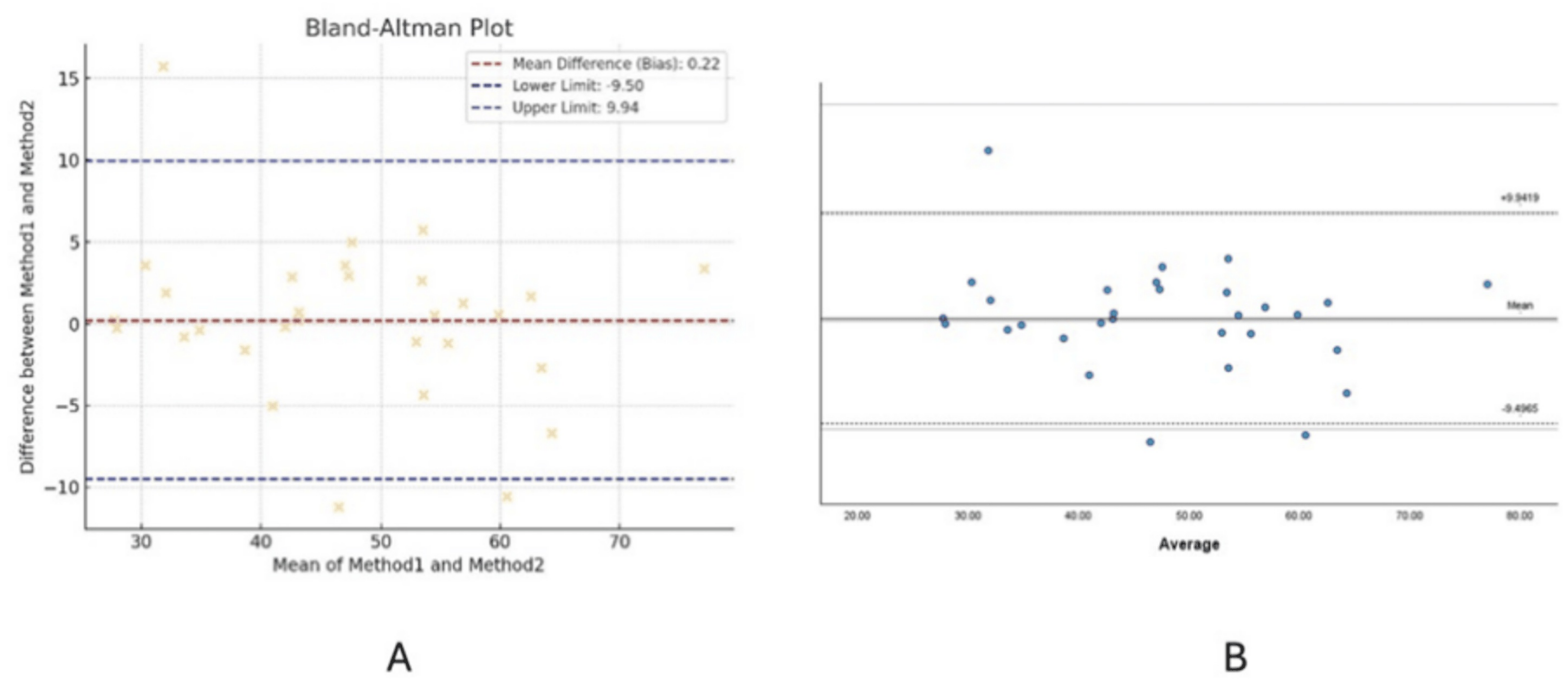
The plot on the right represents the results from ChatGPT-4, while the plot on the left represents the results from SPSS A: ChatGPt-4; B: SPSS

The intraclass correlation coefficient (ICC) analysis showed similar conclusions regarding the level of agreement, with both tools indicating poor agreement. However, differences were noted in the specific ICC values and the 95% confidence intervals (Table 12). In the Bland-Altman analysis, the mean bias was consistent between SPSS and ChatGPT-4, but there were differences in how the 95% limits of agreement were reported (Table 13). Despite this, the Bland-Altman plots were visually identical, showing no significant discrepancies (Figure 1).

**Table 12:** Comparison of intraclass correlation coefficient (ICC) analysis between SPSS and ChatGPT-4 * Differences reported between SPSS and ChatGPT-4.

ICC (95% CI) (lower, upper)	Strength of Agreement	P value
SPSS		
-0.175 (-0.371, 0.259)	Poor Agreement	0.817
ChatGPT		
-0.1174 (-0.0205, -0.2347)*	Poor Agreement	0.817

**Table 13:** Comparison of Bland-Altman plot between SPSS and ChatGPT-4 Kappa statistics: Count (% within rater 1). * Differences reported between SPSS and ChatGPT-4.

N	Mean Bias	Std. Deviation	95% CI (Lower, Upper)	95% Limits of Agreement (Lower, Upper)
SPSS				
30	0.2227	4.9588	2.0744, -1.6289	9.9419, -9.4965,
ChatGPT				
30	0.2227	4.9588	-1.6289*,-2.0744*	-9.4965*, 9.9419*

In the sensitivity and specificity analysis, both tools provided identical sensitivity and specificity values, confirming consistent findings in distinguishing between true positives and true negatives (Table 14). The AUC analysis also showed consistency in AUC values between SPSS and ChatGPT-4, although slight differences in the standard errors and confidence intervals were observed (Table 15).

**Table 14:** Comparison of sensitivity and specificity between SPSS and ChatGPT-4 TP = True Positive; FP = False Positive; FN = False Negative; TN = True Negative

TrueLabel, N (%)	PredictedLabel, N (%)	
SPSS	Yes	No
Yes	28 (TP)	28 (FP)
No	20 (FN)	24 (TN)
Sensitivity, TP/(TP+FN):	0.58	
Specificity, TN/(FP+TN):	0.46	
ChatGPT		
Yes	28 (TP)	28 (FP)
No	20 (FN)	24 (TN)
Sensitivity, TP/(TP+FN):	0.58	
Specificity, TN/(FP+TN):	0.46	

**Table 15:** Comparison of area under the ROC curve (AUC) between SPSS and ChatGPT-4 * Differences reported between SPSS and ChatGPT-4.

Area	Std. error	95% CI (lower, Upper)	P value
SPSS			
0.523	0.058	0.408, 0.637	0.697
ChatGPT			
0.523	0.050*	0.425*, 0.621*	Unable to perform calculations

## Discussion

Comparison of SPSS and ChatGPT-4 in statistical analyses

This study aimed to evaluate the accuracy between traditional statistical software (SPSS) and the AI-based tool ChatGPT-4 across various statistical analyses. In several analyses, including the Independent sample t-test, paired t-test, Pearson correlation, simple linear regression, and the analysis of sensitivity and specificity, both SPSS and ChatGPT-4 produced identical results. These tests demonstrated complete consistency in key outputs such as mean values, standard deviations, correlation coefficients, regression coefficients, p-values, and confidence intervals.

Discrepancies in analyses

However, differences emerged in more complex analyses. The one-way ANOVA analysis revealed significant differences among the groups, with SPSS and ChatGPT-4 delivering consistent statistical results. A notable discrepancy occurred in the post-hoc analysis performed by ChatGPT-4's AI. The AI changed the order of the group comparisons, leading to altered values for the 95% confidence intervals. Specifically, while the SPSS analysis calculated the mean difference as Group 1 minus Group 2, resulting in a negative mean difference, ChatGPT-4's output reversed the subtraction order, yielding positive values. This reversal also affected the confidence intervals, swapping the upper and lower bounds. Such changes in sequence and value could potentially cause confusion in interpreting the results, emphasizing the importance of carefully reviewing AI-generated outputs to ensure clarity and consistency with traditional statistical tools such as SPSS [10].

In the comparison of Pearson’s chi-square test results between SPSS and ChatGPT-4 [11], a significant difference was observed in the statistical methods reported. SPSS highlighted the likelihood ratio, while ChatGPT-4 employed the continuity correction. This difference in reporting reflects the tools' emphasis on different statistical outputs. However, when the research team checked SPSS's output for the continuity correction, it yielded the same value as ChatGPT-4, indicating that the discrepancy was due to the different values reported by each tool rather than a fundamental difference in the statistical calculation.

The differences observed in the Mann-Whitney U test results between SPSS and ChatGPT-4 can be attributed to the distinct methods used for calculating the IQR [12]. SPSS, by default, uses Tukey’s Hinges method for IQR calculation, which is a well-established approach. However, other software environments, such as ChatGPT-4, might use alternative methods. For instance, if ChatGPT-4 used NumPy for calculations, the IQR might have been computed using the type 1 linear method. This variation in IQR calculation methods can lead to differences in the Z-value, as different software programs apply distinct rules in their computations.

Similarly, in Spearman’s correlation [13] and kappa statistics analyses [14], discrepancies arose due to different data handling methods. In SPSS, the default method ensures consistency, but ChatGPT-4's different approaches to managing ties, missing data, or edge cases could lead to minor differences in output. Even small rounding differences during intermediate calculations can impact the final p-value, particularly in small datasets or when the marginal distributions are nearly identical.

For the ICC analysis, the variation between SPSS and ChatGPT-4 could stem from the different methods used for calculation [15]. SPSS offers several models for calculating ICC, such as one-way random effects, two-way random effects, and two-way mixed effects models. These methods vary based on how they account for variability between subjects and raters, leading to differences in reported ICC values between SPSS and ChatGPT-4.

In the Bland-Altman analysis [16], the discrepancy between SPSS and ChatGPT-4 can be explained by how each tool calculates the mean difference. In SPSS, the mean difference was calculated by subtracting Method1 from Method2, leading to a correctly indicated confidence interval. However, if ChatGPT-4 reversed the subtraction order (Method2 minus Method1), the confidence interval would also be reversed, potentially causing the observed discrepancy. This highlights the importance of verifying the direction of subtraction when interpreting Bland-Altman plots, particularly when using different software tools.

Impact of discrepancies 

The observed differences between ChatGPT-4 and SPSS in post-hoc analyses, p-values, interquartile ranges, z-values, likelihood ratios, and confidence intervals could have led to varying interpretations, potentially influencing clinical or research conclusions. Discrepancies in post-hoc analysis methods or the presentation of results may have affected the identification of statistically significant differences between groups, which could have influenced decisions regarding treatment efficacy or group comparisons.

Even small variations in p-values could have determined whether a result was deemed statistically significant, potentially altering the direction of further research or clinical actions. Z-values, used in hypothesis testing and confidence interval calculations, may also have differed between the two tools, leading to different assessments of data significance.

Variations in IQRs, reflecting the spread and variability of data, may have led to different interpretations of data distribution and the identification of outliers, thus influencing conclusions about data quality and the reliability of findings. Differences in likelihood ratios between ChatGPT-4 and SPSS could have affected how relationships in categorical data were interpreted, impacting decisions based on diagnostic or predictive analyses.

Furthermore, confidence intervals, which indicate the range within which the true population parameter is likely to fall, were critical for evaluating the precision and reliability of estimates. Differences in confidence intervals reported by ChatGPT-4 and SPSS -whether narrower or wider - could have altered conclusions about the clinical significance of an effect or the overall confidence in study findings. Such variations might have influenced researchers' interpretation of results, particularly when assessing the uncertainty associated with key estimates.

Comparison to previous studies

In a study comparing ChatGPT-4's performance in biostatistical problem-solving, Ignjatović et al. primarily focused on evaluating ChatGPT-4's abilities using questions from the "Oxford Handbook of Medical Statistics" [5]. When ChatGPT-4 provided incorrect answers, the authors intervened by supplying corrections, allowing the AI to refine its responses through multiple attempts. This iterative process demonstrated ChatGPT-4's ability to improve with guidance, ultimately producing accurate results. However, this approach required continuous adjustments to achieve correct outcomes, highlighting the dependency on user intervention [17].

In contrast, our study assessed ChatGPT-4's initial performance using raw data and directly compared the results with SPSS. Our objective was to evaluate ChatGPT-4's ability to independently generate accurate results without intervention. When discrepancies arose between ChatGPT-4 and SPSS, we tested the AI's response by providing SPSS outputs and asking it to recalculate. While ChatGPT-4 occasionally adjusted its results to align with SPSS, it sometimes altered other values in the process [18], raising concerns about consistency and reliability [19,20]. Unlike the previous study where the AI refined its outputs, ChatGPT-4 often insisted on the correctness of its initial calculations in our study, even when inconsistencies were apparent, attributing differences to variations in data handling between the tools.

This hands-off approach underscores the importance of verifying the entire output from ChatGPT-4 rather than relying solely on a single correct value. The importance of verifying AI-driven outputs is further emphasized by findings from another study conducted by Huang et al., which compared statistical analyses across ChatGPT-4, SPSS, R, and SAS [4]. This previous study categorized differences between these tools, highlighting categorical similarities but not specifying the exact aspects of descriptive statistics, ANOVA, or correlation analyses being compared. This approach can obscure crucial details, especially in fields where minor variations can significantly impact results.

In contrast, our study took a more precise approach by directly comparing ChatGPT-4's outputs to those generated by SPSS without categorizing the results. By measuring exact differences in statistical outputs, such as the F-value in ANOVA or specific descriptive statistics, our study revealed that, while certain values were highly consistent between ChatGPT-4 and SPSS, others showed inconsistencies. These findings underscore the importance of not relying solely on categorical similarities but instead ensuring that each individual value is verified, particularly in research areas where precision is paramount.

Data privacy and ethical considerations

Additionally, the author would like to emphasize another critical issue related to the use of ChatGPT-4 in research - data privacy [21]. Since ChatGPT-4 tailors its responses based on interactions with users, there is a possibility that research data entered into the system could be stored on its data servers, raising concerns about potential data breaches, particularly with sensitive or confidential research data. During the ethical approval process, researchers should disclose the use of AI tools such as ChatGPT-4 and outline the associated risks. Transparency about data handling is essential to ensure ethical guidelines are followed, particularly concerning data privacy. Although OpenAI allows users to disable chat history, ensuring that data are only stored for 30 days and reviewed only in cases of abuse, this measure may not be sufficient for highly sensitive research data.

Institutions should provide clear guidelines on the use of AI in research. For instance, the University of Wollongong in Australia advises that data subject to privacy legislation, such as identifiable human data or private/personal information, should not be entered into generative AI tools [22]. By following such guidelines, researchers can better protect sensitive data and mitigate risks associated with AI-driven research tools. Ensuring that all potential risks are addressed during the ethical approval process is essential to maintaining the integrity and privacy of research data.

Furthermore, an ethical issue that required attention was the potential for data manipulation by AI. With the ease at which AI handled large datasets and performed multiple calculations within seconds, manipulating data to achieve desired outcomes became much simpler. ChatGPT, for example, had the capability to modify raw datasets to produce a desired statistical significance, such as a specific p-value, which raised concerns about the integrity of research outcomes. In response to this, there should not only be tools to detect AI involvement in the writing of manuscripts but also tools specifically designed to detect AI manipulation of raw datasets. Such safeguards are essential to ensure transparency, maintain the validity of scientific findings, and protect the integrity of research.

Performance and usability considerations

Although ChatGPT-4 can quickly analyze data, its inconsistencies can delay the completion of work, particularly when errors occur. For example, during this study, ChatGPT-4 occasionally failed to process the dataset, displaying messages such as "It looks like there was an issue accessing the uploaded file. Could you please try uploading the file again?" and "Unable to display visualization." These intermittent disruptions persisted for several hours at times, highlighting the inconsistent performance of ChatGPT-4 and its potential to delay work.

While ChatGPT-4 offers great value due to its ease of use and speed in performing analyses [23], fully utilizing its capabilities, particularly when integrated with Python, requires a deep understanding of statistical procedures and data handling methods. Unlike SPSS, which provides a user-friendly interface with transparent assumptions and calculations, ChatGPT-4’s reliance on Python code can make it challenging to verify and interpret the details of its outputs [24]. This complexity may be a barrier for those less familiar with programming, making SPSS more suitable for detailed analyses where understanding underlying assumptions is crucial. To address this, one potential strategy is to use ChatGPT-4 for generating instructions on performing analysis in SPSS, which may be a better alternative for beginner users. If inaccuracies arise, SPSS's transparent outputs make it easier to detect errors compared to interpreting Python code generated by ChatGPT-4. Additionally, as new versions of ChatGPT are released, it is likely that its reliability and performance in statistical analysis will improve, enhancing its utility for future research.

Implications to medical and dental research 

In medical and dental research, ChatGPT-4 can be a useful tool for data analysis, but clinicians and researchers must understand its limitations. It should be treated similarly to a human rater in a study, where calibration and reliability checks are necessary before using it on raw data. Ensuring that prompts are clear for the AI to interpret correctly is crucial. Additionally, inter-rater reliability should be assessed to confirm that ChatGPT-4 produces consistent results when compared to other data analysis tools. Once ChatGPT-4 has been calibrated and its reliability established, researchers can use it more confidently.

Limitation of study

This study had several limitations. First, it focused on a specific set of statistical analyses, which may not fully capture potential discrepancies that could arise with more advanced or complex methods. The datasets used were relatively limited in size and complexity, meaning the performance of both ChatGPT-4 and SPSS with larger or more nuanced datasets was not thoroughly tested. Additionally, ChatGPT-4's performance occasionally exhibited variability, including processing delays, which could affect its reliability for time-sensitive analyses.

A key limitation was that ChatGPT-4's handling of the dataset was highly dependent on the clarity and precision of the user's input. The AI required direct and unambiguous instructions, and variations in the prompts could lead to different outcomes. Furthermore, this study did not conduct a detailed analysis of how ChatGPT-4 responded to corrections or adjustments in the instructions. While ChatGPT-4 had the capability to modify its outputs based on additional guidance, this may have resulted in selective alterations that could create discrepancies when attempting to align its outputs with those of SPSS. This aspect of ChatGPT-4’s accuracy was not thoroughly examined in the present study.

## Conclusions

ChatGPT-4 produced accurate results for many statistical tests, aligning well with SPSS for simpler analyses, such as the independent t-test, paired t-test, and simple linear regression. However, discrepancies in post-hoc analyses, confidence intervals, and more complex tests such as the Mann-Whitney U and Wilcoxon signed-rank tests indicate that, while ChatGPT-4 has potential, its use in complex statistical analyses requires careful validation. Researchers should exercise caution, particularly for tests involving detailed calculations, and ensure results are cross-validated with established tools such as SPSS.
